# COVID-19: Precautionary Guidelines for Ophthalmologists

**DOI:** 10.7759/cureus.8815

**Published:** 2020-06-25

**Authors:** Hani B ALBalawi

**Affiliations:** 1 Ophthalmology, University of Tabuk, College of Medicine, Tabuk City, SAU

**Keywords:** covid-19, conjunctivitis, prevention ophthalmology, ophthalmology, guideline, infection, health care worker safety, corona pandemic, coronavirus guidelines, coronavirus

## Abstract

Several coronaviruses can infect humans, and the globally endemic human coronaviruses, HCoV-229E (human coronavirus 229E), HCoV-NL63 (human coronavirus NL63), and others, tend to cause mild respiratory diseases. The zoonotic Middle East respiratory syndrome coronavirus (MERS-CoV) and severe acute respiratory syndrome coronavirus type1 (SARS-CoV-1) have high fatality rates. In December 2019, the World Health Organization (WHO) was notified by Chinese authorities about an outbreak of pneumonia before the causative organism was identified in January 2020 as a novel coronavirus family. The WHO refers to the virus as coronavirus disease 2019 (COVID-19). Within several weeks, the outbreak has become an emergency, and many countries have since been affected. The method of transmission is not yet fully known but is thought to be mainly respiratory. Healthcare providers, particularly ophthalmologists, are at high risk of a COVID-19 infection through unprotected contact with eye secretions during routine ophthalmic examinations that involve the use of direct ophthalmoscopy and slit-lamp examinations, which are usually performed in a setting that allows for close doctor-patient contact. In light of these, specific measures are needed from an ophthalmic point of view to control the COVID-19 outbreak and to protect health care providers.

## Introduction

A 33-year-old ophthalmologist, Dr. Li Wenliang, was the first to raise the alarm about a cluster of severe acute respiratory syndrome (SARS)-like pneumonia cases late December 2019 at Wuhan City, China. Regrettably, that warning was not taken seriously. Following that, Dr. Li returned to work and, unfortunately, contracted the virus from an asymptomatic glaucoma patient in early January. Later on, Dr. Li passed away, leaving behind a child and a wife, who is pregnant, and opened the doors and discussions about the risk of infection among ophthalmologists. A survey study by Nair and colleagues reported that the majority of ophthalmologists in India (59.1%) felt that ophthalmologists were potentially at a higher risk of contracting COVID-19 as compared to other specialties while examining patients [1]. In fact, ophthalmologists are at high risk of contracting the COVID-19 virus through unprotected eye contact with secretions during routine ophthalmic examinations with direct ophthalmoscopy and slit-lamp examinations, which are usually performed in a setting that has close doctor-patient contact. On December 31, 2019, the WHO was notified by China about an outbreak of pneumonia with an initially unknown etiology in Wuhan city in Hubei province. In January 2020, the causative organism was identified as a novel member of the coronavirus family. Initially tentatively named 2019 novel coronavirus (2019-nCoV) and named SARS-CoV- type 2 by the International Committee of Taxonomy of Viruses (ICTV), this virus is the cause of the disease named coronavirus disease 2019 (COVID-19). To date, COVID-19 has already confirmed to have affected over 5,000,000 people and caused over 300,00 deaths worldwide, spanning 205 countries in South East Asia, Europe, North America, Australia, and the Middle East and led to huge public health challenges [2].

The transmission route of COVID-19 is not yet fully known but it is thought to be mainly respiratory. It is even more overwhelming that many infected persons showed no apparent respiratory symptoms and presented with non-respiratory manifestations, including conjunctivitis in the ocular tissue [3] and diarrhea in the gastrointestinal system [4]. COVID-19 may spread through the eye’s mucous membrane via indirect contact with droplets. The variable presentation of the disease makes transmission via contact, especially for health care providers like ophthalmologists, possible. Since the outbreak, more than 3,300 healthcare professionals having been infected, with 44 deaths, including one ophthalmologist. Multiple comprehensive recommendations by the American Academy of Ophthalmology (AAO) [5] and a well-designed study from Hong Kong [6] were announced helping ophthalmologists for safe practices. However, it is the responsibility of global organizations to review daily evidence and provide instructions and uniform guidelines. This paper aimed to provide an evidence-based approach, although this may not be applicable to all institutes and is not a proven medico-legal recommendation. Subsequent information about the nature of COVID-19 may change these recommendations in the future. In light of these, specific measures are needed from an ophthalmic point of view to control the COVID-019 outbreak and to protect health care providers.

## Technical report

Before encountering patients during a pandemic lockdown

Because of the disease's contagiousness and way of spread, it is better and safe to keep non-urgent patients away from the hospital during the pandemic period. The priority should be on how to reduce the risk of COVID-19 virus transmission from human to human and the rate of new case development. Then, we “flatten the curve” and not overwhelm the health services and limited supply of hospital beds, intensive care unit (ICU) beds, ventilators, and others. Second, direct all disposable medical supplies and focus them on hospitals where they are most needed [5]. Therefore, all non-urgent routine follow-up and surgeries like mild non-proliferative diabetic retinopathy or routine scheduled cataract surgery should be postponed. It is even better if patients can be sent text messages or called to discuss the need to reschedule their appointment or a public announcement to cancel all routine follow-up visits and surgeries, in order to minimize all non-urgent cases coming to the eye clinic or hospital [5]. A three-stage control measure to reduce the transmission of the virus in the ophthalmology department in Hong Kong was based on text messaging to reschedule refill visits [6]; a triage to identify patients with fever, conjunctivitis, and respiratory symptoms; asking those who recently traveled to areas infected with the virus to postpone their ophthalmology visits for 14 days; and the avoidance of micro-aerosol generating procedures, nasal endoscopy, and operations under general anesthesia. In addition, infection control was provided to the environment and health personnel.

When facing patients during the pandemic lockdown

**Figure 1:**
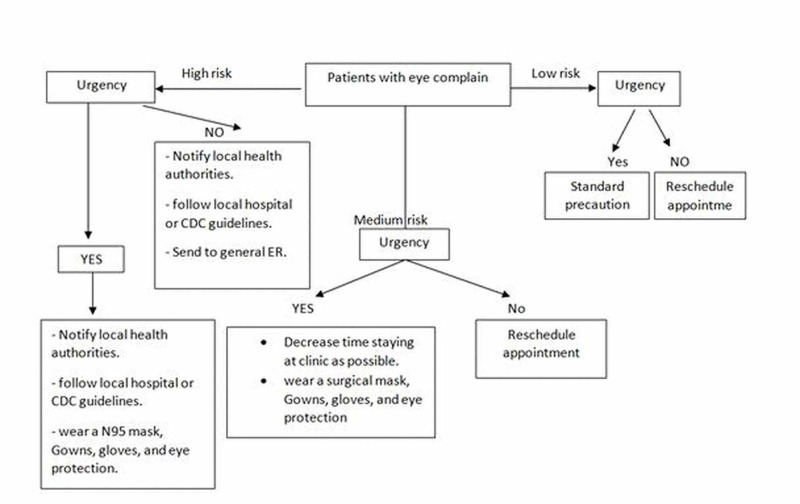
How to deal with patients during the COVID-19 pandemic

Always keep in mind that the main goal during this pandemic is to protect yourself and your patient, preserve all health services to stop its spread, and prevent the health system from being compromised. It is safer to divide patients who attend eye clinics or have ophthalmology complaints into three groups: low risk, medium risk, and high risk (Table 1) [7]. Ophthalmology clinics that see patients should be equipped with screening stations before entry to the waiting area and must take a detailed history about any respiratory illnesses, fever, recent return from high-risk areas, or contact with family members recently back from one of the countries battling a COVID-19 outbreak [5]. This way, every patient can be classified as low, medium, or high risk. This should be coupled with nurse-directed triage protocols to determine if an appointment is necessary or if the patient can reschedule and be sent home to ensure safe triage and isolation of patients presenting with COVID-19 symptoms or other respiratory infections. Those at low risk can be attended to with standard precautions. Medium-risk patients with non-urgent ophthalmic problems should be asked to return home and reschedule their appointment [5]. High-risk patients with a non-urgent ophthalmic problem should be sent to the emergency room (ER) immediately and local health authorities notified. Medium-risk patients with urgent ophthalmic problems should be put in the examination lane immediately in order to decrease the time spent at the clinic. The patient, the ophthalmologist, and all health care personnel should wear surgical masks, gowns, gloves, and eye protection. In case of any planned procedure that results in an aerosolized virus, an N95 mask should be worn. High-risk or COVID-19 positive patients with urgent ophthalmic problems can be seen but after following local hospital guidelines and precautions for treating patients with COVID-19 or that of the Centers for Disease Control and Prevention (CDC) such as using an N95 mask, eye protection, and gowns (Figure 1) [5]. Standard precautions for every patient should be followed, including using a breath shield during a slit-lamp exam or any clear plastic barrier to block the transfer of breaths between the patient and the doctor (2). Ophthalmologists should tell their patients that they will speak as little as possible during the slit-lamp examination and request that the patients also refrain from talking. They should always use protection for the mouth, nose, and eyes when caring for patients potentially infected with COVID-19. They should also keep the waiting room as empty as possible and advise seated patients to remain at least two meters (around 6 feet) from one another [5]. As much as is prudent, they should reduce the visits of the most vulnerable patients. Patients with or suspected to have COVID-19 should be cared for in a single-person room with the door closed. Limited points of entry to the facility is strongly recommended too.

**Table 1:** Risk groups and symptoms of possible SARS-CoV-2 (COVID-19) infection in patients seeing at an ophthalmology clinic SARS-CoV-2: severe acute respiratory syndrome coronavirus 2 [7]

Low-risk	No symptoms (e.g, cough, fever, breathlessness, diarrhea. No contact with someone who is SARS-CoV-2 positive. No stay in a high-risk area during the previous 14 days
Medium-risk	Presence of symptoms with: No medical history or contact with someone who is SARS-CoV-2 positive. No stay in a high-risk area during the previous 14 days
High-risk	At least one symptom out of the following: Contact with someone who is SARS-CoV-2 positive. Stay in a high-risk area during the previous 14 days

Reopening the ophthalmology clinic

**Figure 2:**
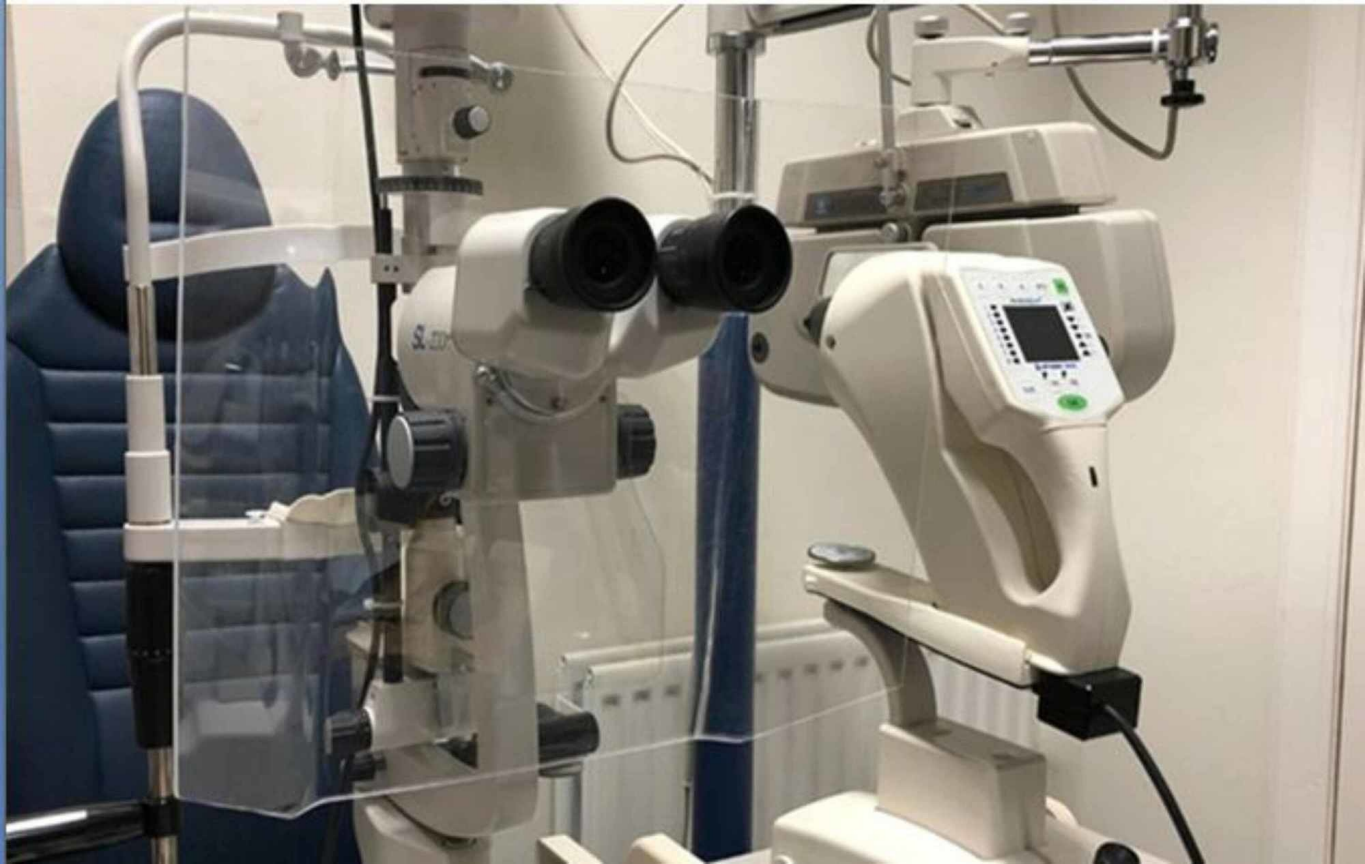
Plastic barrier to block the transfer of breaths between the patient and the doctor

Before opening a routine clinic, checklist criteria that aim at safe opening should be available. Complying with local governmental guidance, these guidelines should be closely reviewed and followed. Making a plan and pre-opening planning will be critically important to the success of your practice reopening, including an assessment of your personal protective equipment (PPE) needs and alternatives such as cloth masks, eye protection, and others [8]. Evaluate the clinics to determine what kinds of surfaces and materials make up that area, as most surfaces just need normal, routine cleaning; however, frequently touched surfaces like doorknobs will need to be cleaned with soap and water and then disinfected using United States Environmental Protection Agency (EPA)-approved disinfectant. If a disinfectant is unavailable, you can use one-third cup of bleach added to 1 gallon of water or 70% alcohol solutions to disinfect. Make sure all slit-lamps are equipped with a breath shield (Figure 2). It is better that staff who do not need to be physically present in the office stay at home and work remotely. Even more, consider bringing employees back in stages, at different parts of the day, or working on alternative days. Start with a few outpatients visits a day and work on a modified schedule to avoid high volume or density. Unless necessary, ask patients to attend alone, keep the waiting room as empty as possible, and advise seated patients to remain at least two meters (around 6 feet) from one another, consider another option to wait in the clinic by asking patients to wait in their car or outside and text or calling when the appointment is ready. Remove magazines, brochures, remote controls, and other shared items from waiting and exam rooms. Follow U.S. Centers for Disease Control and Prevention (CDC) guidance, which requires all individuals who visit the office to wear a cloth face covering. This should be explained to patients and other visitors before they arrive at the clinic. Prioritize seniors above 60 years, those with chronic diseases like diabetes mellitus, and patients on chemotherapy to be seen and leave the clinic early. The availability of screening stations before entry to the waiting area should also continue to pick up any suspected cases and then deal with them as high-risk patients.

After seeing a patient

After seeing every patient, standard cleaning and disinfection should apply to all patient care, regardless of the suspected or confirmed infection status of the patient [5]. Wear single-use gloves when cleaning and disinfecting surfaces. All ophthalmic instruments, including slit-lamps, tonometer, and contact lenses, and all places the patient touched should be disinfected. Standard hand hygiene is critical, including frequent hand wash with soap and water for at least 20 seconds before and after seeing patients [8]. If soap and water are not readily available, use a hand sanitizer that contains at least 60% alcohol [5]. The virus causing COVID-19 is an enveloped virus, making it very susceptible to the same alcohol- and bleach-based disinfectants that ophthalmologists commonly use to disinfect ophthalmic instruments and office furniture. Some methods recommended by the CDC to disinfect and destroy the COVID-19 virus include alcohol solutions with at least 70% alcohol and diluted bleach. However, the United States Environmental Protection Agency (EPA) shared a list of antimicrobial products like Stepan Company (Northfield, Illinois) products (disinfectant spray) that are expected to be effective against COVID-19 [9]. The importance of disinfecting ophthalmic examination instruments to minimize cross-infections and protect ophthalmic medical staff is clear. There are a wide range of disinfectants available that can be used to disinfect surfaces. Kampf et al. found that some members of the coronavirus family - SARS and Middle East respiratory syndrome (MERS) - can persist on surfaces like metal, glass, or plastic for up to nine days but can be efficiently inactivated by surface disinfection with 62%-71% ethanol, 0.5% hydrogen peroxide, or 0.1% sodium hypochlorite within one minute. Other biocidal agents, such as 0.05% to 0.2% benzalkonium chloride or 0.02% chlorhexidine digluconate, are less effective [10].

## Discussion

COVID-19 infection and possible eye involvement

Severe acute respiratory syndrome coronavirus type 2 (SARS-CoV-2), also known as COVID-19 virus, is a species of the coronavirus family that infects humans, bats, and certain other mammals. In fact, bats are a major reservoir of many strains of SARS-related coronaviruses, and several strains have been identified in palm civets, which were likely ancestors of SARS-CoV-2. Cross-species transmission (CST), also called host jump, or spillover, is the ability of a foreign virus, once introduced into a new host species, to infect that individual and spread throughout the new host population, as has happened with swine flu, SARS-COV-1, and now COVID-19 [11]. It is a member of the genus betacoronavirus and subgenus Sarbecoronavirus and is an enveloped positive-sense single-stranded RNA virus that enters its host cell by binding to the angiotensin-converting enzyme 2 receptor (ACE2) receptor [12]. Human to human transmission occurs with close contact with an infected person. This primarily occurs when an infected person coughs or sneezes and produces respiratory droplets that spread like other respiratory pathogens. These droplets can settle in the mouth or nasal mucosa and lungs of people who inhale the air [13]. However, it is widely agreed that the clinical picture varies from a simple respiratory infection, which presents with symptoms such as a runny nose, sore throat, fever, and coughing to more complicated findings like septic shock [14]. Similar to SARS-CoV and MERS-CoV, which caused epidemics in the past years, the usual first symptoms are commonly defined as fever, cough, and shortness of breath. In 99 patients with COVID-19 virus infection, chest pain, confusion, and nausea-vomiting were noted in addition to previous findings [15]. X-ray and thorax CT imaging of patients with COVID-19 showed unilateral or bilateral involvement compatible with viral pneumonia. Reported complications include acute heart damage, acute respiratory distress syndrome (ARDS), secondary infections, pneumothorax, and some patients with other comorbidity exhibited more severe symptoms and even death [16]. The diagnosis of COVID-19 infection is based on history, including detailed contact and travel history aided by laboratory testing. Viral infection laboratory testing methods are important, including serology tests and viral culture. The most commonly used diagnostic methods are molecular methods, such as reverse transcription-polymerase chain reaction (RT-PCR) or real-time PCR by using a respiratory sample as the oropharyngeal swab, sputum, or bronchoalveolar lavage.

Unfortunately, the COVID-19 virus not only affects the respiratory tracts, it also may manifest in other regions like the gastrointestinal tract and ocular tissues, as conjunctivitis has been reported during this outbreak and conjunctival secretions yielded positive RT-PCR results [17]. This suggests that COVID-19 can infect the conjunctiva and cause conjunctivitis, and virus particles are present in ocular secretions. Indeed, there is also evidence that some coronavirus family can occasionally cause conjunctivitis in humans. In fact, human coronavirus NL 63 (HCoV-NL63) was first identified in a baby with bronchiolitis and conjunctivitis. In 28 cases of children with confirmed HCoV-NL63 infections, 17% had conjunctivitis [18]. Clinical entities, such as conjunctivitis, anterior uveitis, retinitis, and optic neuritis, have been seen in animal models [19]. One of the affected healthcare workers reported his experience of the disease. Despite being fully dressed in a protective suit and N95 respirator, he was still infected by the virus, with the first symptom being unilateral conjunctivitis, followed by fever. In spite of the controversy about the eye as the possible means of transmitting disease, Qing et al. posit that the virus enters the tear duct through droplets, which may pass through the nasolacrimal ducts and then into the respiratory tract [20]. Combined with all this information, we assert that healthcare professionals should use eye protection when in close contact with patients.

## Conclusions

At the time of the pandemic, with limited information, no treatment for COVID-19, and no vaccine as yet, we will need to stay highly cautious to recognize the possible non-respiratory early manifestations of COVID-19 infection, including the consideration of viral conjunctivitis as a possible presentation. Healthcare professionals, including ophthalmologists, should take the full recommended measures and precautions to protect themselves and their patients and stop the spread of the infection.
